# The role of community resilience as a protective factor in coping with mental disorders in a sample of psychiatric migrants

**DOI:** 10.3389/fpsyt.2024.1430688

**Published:** 2024-08-08

**Authors:** Martina Olcese, Francesco Madera, Paola Cardinali, Gianluca Serafini, Laura Migliorini

**Affiliations:** ^1^ Department of Education Sciences, University of Genoa, Genoa, Italy; ^2^ Department of Economics, Mercatorum University, Rome, Italy; ^3^ Department of Neuroscience, Rehabilitation, Ophthalmology, Genetics, Maternal and Child Health, Section of Psychiatry, IRCCS Ospedale Policlinico San Martino, University of Genoa, Genoa, Italy

**Keywords:** mental disorders, migration, perceived community resilience, subjective wellbeing, protective factor

## Abstract

**Background:**

Over the past decade migration to Italy has increased significantly for various reasons including armed conflicts. Generally, the migration process is exposed to different risk factors during different periods of migration, which can compromise well-being and promote the onset or exacerbation of mental disorders. A community with resources and the perception of one’s community as resilient can be important protective factor in the context of migration.

**Purpose:**

This study aims to understand which variables in migration predict an increase in perceived community resilience and to understand the role of community resilience in the relationship between mental disorders and subjective well-being in a sample of 100 adult migrants at the first consultation interview in the ambulatories of Psychiatry Unit.

**Methods:**

After defining the inclusion and exclusion criteria, migrants were asked to fill out self-report questionnaires to collect socio-demographic data and to assess perception of mental disorders, perceived community resilience and perception of subjective well-being. Descriptive analysis, simple regression, and moderation analyses were conducted to test the hypotheses.

**Results:**

The results show that the variable meaning attributed to the community with reference to the host community, migration with someone, and longer duration of stay in Italy contribute to increased perceptions of community resilience. In addition, a direct negative effect of mental disorders on subjective well-being and the moderating role of community resilience in relationship between mental disorders and subjective well-being have been demonstrated.

**Conclusions:**

This result underscores the importance of perceived community resilience in mitigating the negative effects of mental disorders on subjective well-being. Perceiving one’s community as more resilient seems to protect against the impact of mental disorders on subjective well-being. Our results support an ecological model of migrants’ mental health that values the community and its resources in coping with mental disorders in the context of migration.

## 1 Introduction

Over the past 15 years, international migration has increased to include approximately 272 million people, or 3.5% of the world’s population. These migrants are unevenly distributed among and within countries (1). In the past ten years, the number of migrants coming to Italy peaked in 2016, with 181,000 individuals. In 2023 150,000 migrants arrived in Italy, 50% more than in 2022, and today they represent 7.4% of the Italian population (2). The reasons people migrate are diverse and involve personal, social, and professional motivations as well as different age groups (3). Recent literature emphasizes environmental disasters such as earthquakes or hurricanes, wars, and climate change among the causes of migration, calling the latter environmental migration (4). Finally, people may migrate individually, with family members, or with other people from their ethnic communities (5).

Although individuals migrate for various reasons, the migration process always presents significant challenges, requiring personal and collective adaptations for both voluntary and forced migrants (6). Migration experiences can also expose people to various traumatic factors, classified according to the migration period (7). In the pre-migration period, people may experience violence, loss of family members, or disruption of family and community life; the transit period may be characterized by difficult and strenuous travel conditions; and finally, in the post-migration period, once migrants arrive in the host country, they may experience various difficult experiences, including stigma, isolation, language barriers, and employment difficulties (8).

These periods, are shaped by various individual and community factors, including the possibility of relying on the presence of someone who can help one feel supported and reduce the impact of migration stressors (9). Similarly, once they arrive in the host context, migrants go through further stages, which are: stability on arrival, psychological destabilization, exploration and re-stabilization, and finally return to normal life. In the re-stabilization phase, which is accessed because of the increased time spent within the host context (10, 11), migrants acquire more information about the host culture and create more bonds, and the host community is perceived as an appropriate place for everyday life.

The concept of community can be described as a collection of individuals characterized by diversity and close social ties who share common visions and participate in collective actions within defined geographic contexts or environments (12). Referring to the host community may reflect a feeling of being integrated into it, being able to perceive access to its resources, having sufficient information about services in the area, and having a support network (13–15). Migrants may consider their community both the one to which they ethnically belong and the host context (16). As previously mentioned, migration can expose people to different traumatic factors, which can compromise their mental health and well-being and may increase their risk of developing psychiatric disorders, including anxiety disorders, mood disorders, post-traumatic stress disorder (PTSD), psychosis, and substance use disorders (17, 18). The data also suggest that migrants are at a higher risk of developing mental disorders than the host population (19).

In Italy, a recent study reported that the number of migrants requiring psychiatric care increased from 2.5% in 2000 to 14% in 2015, mainly in young male individuals with psychosis (20). Psychopathology, in general, has been studied in the literature as strongly negatively associated with perceived well-being (21, 22). Well-being involves satisfaction with life as a whole and with specific areas, such as health, economic status, relationships, community, and occupational life (23). Perceptions of greater well-being can promote lower perception of mental disorders and prevent relapse (24). Consequently, recovery should be measured in terms of both reduced psychiatric symptoms and increased well-being (22). This is in line with the World Health Organization’s definition of mental health, which defines it as a state of well-being in which a person is aware of his or her abilities, can cope with normal life stressors, can work productively and fruitfully, and is able to make a contribution to his or her community (25, 26). Thus, mental health comes from both the reduction of mental illness and improvement of well-being in relation to the community dimension (27). In this regard, in the ecological and multilevel approach to migration, the community with its resources can be a protective factor against the traumatic factors to which migrants are exposed (28). The protective aspects of the community include both the migrant and host communities (29). One of the protective factors that contributes to the well-being of individuals is the perceived resilience of the community (30, 31). In an ecological framework, community resilience is a crucial concept that enables communities to mobilize sufficient resources to face challenges, such as migration (16). Several definitions of community resilience exist in the literature. For example, Norris et al. (32) defined it as a process that links a set of adaptive capabilities (i.e. robust, redundant and rapidly accessible resources) to a positive trajectory of functioning after a crisis. Pfefferbaum et al. (33) conceptualize it as the community’s ability to adapt and sustain functionality during disturbances. In general, many factors contribute to the perception of community resilience including social support, community competence, the presence of resources, and information (34). In fact, feeling that one can access community resources, having sufficient information about services in the area or migration-related bureaucracy, and finally feeling that one belongs to the host community and perceiving empowerment are some of the key factors in perceived community resilience in the context of migration (16).

Factors related to community resilience may reduce the perception of mental suffering, such as the perception of depressive and anxiety symptoms. For example, perceived social support contributes to a lower perception of PTSD symptoms (35). Furthermore, community resilience has been shown in the literature to be negatively correlated with anxiety, depression, and somatization and positively correlated with life satisfaction (36). Finally, perception of community resilience appears to play a moderating role in the relationship between perception of anxiety, depressive symptoms and quality of life (37).

Community resilience has been studied in relation to mental disorders in several contexts (22, 38); however, this relationship has not been investigated among migrants. Understanding community resilience among migrant psychiatric patients is crucial because it can provide insights into how social and environmental factors contribute to mental health and overall well-being (16). This study addresses a significant gap in the existing literature, and it is the first to explore the role of perceived community resilience in the relationship between mental disorders and well-being in a sample of migrant psychiatric patients. Furthermore, despite the presence of many migrants in Italy, studies on psychiatry and migration are still poorly implemented in the Italian context (39). This lack of research in Italy highlights the need for focused studies that can inform both policy and practice to better support this vulnerable population. To fill these research gaps, the purpose of the present study was to:

(1) exploring which migration variables (e.g., solitary migration, meaning attributed to the word community and the duration of stay played a role in predicting increased community resilience, and (2) exploring the moderating role of community resilience in the relationship between mental disorder and subjective well-being (40).

Based on this literature analysis, the following hypotheses were developed:

H1: Migrating with someone, ascribing the meaning of the host community to the word “community”, and having spent more years in Italy (i.e., duration of stay in years in the host context) together predict higher perceptions of community resilience.

**Figure 1:**
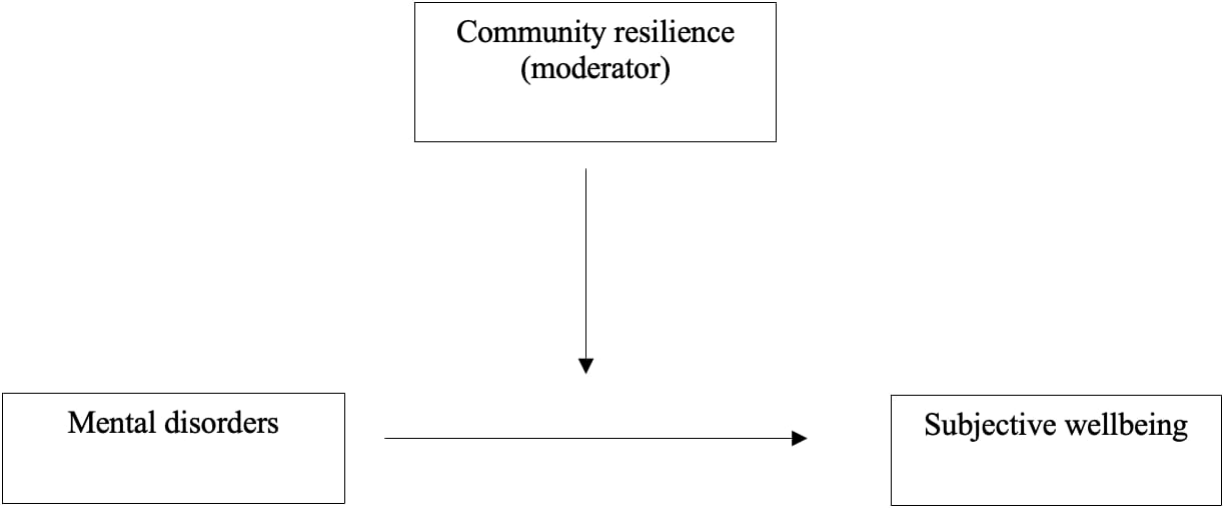
Assumed moderation statistical diagram (Hypothesis 2).

H2: Perceptions of community resilience may moderate the relationship between levels of mental disorder and perceptions of subjective well-being. Specifically, perceptions of community resilience may reduce the negative effects of mental disorders on subjective well-being by acting as a moderator (**Figure 1**).

## 2 Method

### 2.1 Participant

The convenience sample consisted of 100 adult migrant subjects at the first consultation interview at the ambulatories the Psychiatry Unit of Genoa, Italy. The ambulatory clinics engaged in prevention, diagnosis, and support activities through a multidisciplinary team.

### 2.2 Procedure

According to the definition of the World Health Organization (25), we considered migrants to be people who moved from one context to another with different ethnic and cultural characteristics for different periods of time and reasons.

The inclusion criteria for this study were: (a) being migrants, (b) being adults aged over 18 years, and (c) understanding the Italian language to be able to understand the questions and answer them.

Participants were excluded if they had (a) psychopathological decompensation, (b) intellectual disability.

The study was conducted between January 2022 and January 2024, and data were collected during the first consultation interview in the outpatient clinic rooms of the psychiatric clinical unit. The psychiatrists who collaborated in the research and conducted the first interview assessed the eligibility of patients to participate in the study, with reference to the inclusion and exclusion criteria mentioned above. Specifically, they assessed through observation, psychiatric clinical interview, and psychic examination whether psychopathological decompensation - i.e., acute symptoms or exacerbations of pre-existing mental disorders, in accordance with DSM V (41) - or intellectual disability were present that prevented participation in the research. Conversely, if patients were assessed as suitable, they were asked to participate in the study at the end of the interview. Once their willingness was obtained, they were taken to another room where the research and questionnaires were explained to them, and their written informed consent was obtained. In addition, a psychiatrist other than the one who had conducted the first interview was available to provide additional information or clarification that was helpful in completing the self-administered questionnaires.

All participants provided written informed consent before participating in the study. Voluntary participation, confidentiality and anonymity were ensured.

Participants were asked to complete a survey exploring various constructs relevant to the study, as well as an *ad hoc* sociodemographic sheet. The study was approved by the members of the Ethical Research Committee of the University of Genoa approved the project with protocol N. 202335.

### 2.3 Measures

The instruments used in this study were as follows:

*Ad hoc* items were created to evaluate solitary migration (“Did you migrate alone?”), the meaning attributed to the word community (“Does community mean ethnic community to you?” or “Does community mean host context community to you?”) and duration of stay (“How many years have you lived in Italy? Indicate the number of years.”). The first two items provided binary response categories (Yes/No) with code “1” representing the presence of the variable under consideration and “0” the absence. For example, if the answer to the question about alone migration was negative, migration with someone was considered.

Perceived Community Resilience. *Communities Advancing Resilience Toolkit Assessment Survey – Core Resilience* (CART) (42). This self-report questionnaire includes 24 items (e.g., “People in my community help each other”) measuring five domains of perceived community resilience: connection and caring (5 items); resources (5 items); transformative potential (6 items); disaster management (4 items); information and communication (4 items). The items are scored on a 5-point Likert scale with values ranging from 1 (i.e., strongly disagree) to 5 (i.e., strongly agree). The total score ranges from 24 to 120, and a higher score represents a higher level of perceived community resilience. Reliability was excellent (Cronbach’s α = 0.96).

Subjective Wellbeing. *Interpersonal, Community, Occupational, Physical, Psychological and Economic Wellbeing Scale* (I-COPPE) (28). This self-report questionnaire includes 21 items (e.g., “Considering what your relationships with the important people in your life are like these days, which number would you choose?”) drawing on 7 related domains of people’s subjective well-being - Overall, Interpersonal, Community, Occupational, Physical, Psychological, and Economic. Each item (3 for domains) was assessed with reference to present (i.e., in the current moment), to past (i.e., in the previous year), and to future (i.e., in the next year). Participants were asked to rate each item on a scale from 0 (i.e., worst your life can be) to 10 (i.e., best your life can be). The total score ranges from 0 to 210, with a higher score representing a higher level of subjective well-being. Reliability was excellent (Cronbach’s α = 0.95).

Mental Disorders. *Self-Reporting Questionnaire* (SRQ-24) (43). It is a self-report questionnaire developed by the World Health Organization for the healthcare context to evaluate mental disorders. It has been tested in different migrant populations, demonstrating good validity (44, 45) and culturally sensitive nature (46). It consists of 24 items. The first 20 items concern symptoms such as depressive, anxious or psychosomatic (e.g. “Have you lost interest in things?”). The last four items reflect psychotic symptoms (e.g., “Have you noticed interference or anything unusual in your thoughts?”). It includes binary (yes/no) questions only, with codes “1” which represents the perception of presence of a symptom, and “0” if the symptom is not perceived. The total score ranges from 0 to 24, with a higher score representing a higher perception of symptoms of mental disorder. Reliability was good (Cronbach’s α = 0.83).

### 2.4 Data analysis

All data were entered into the Statistical Package for the Social Sciences, version 25.0 (SPSS 25) (47). The significance level of the tests was set at 5%, and all the necessary analyses were performed. First, as the normality tests showed that the data were approximately normally distributed (Shapiro-Wilk *p* < 0.005 and Kolmogorov-Smirnov *p* < 0.005) further parametric tests were performed. Descriptive analyses of the study variables were performed, such as calculating means, standard deviations and frequencies. Bivariate correlation analysis (Pearson’s r) was performed for all variables to observe their association. As all the assumptions required to perform a linear regression were met, a simple linear regression was used to identify the predictors of community resilience among the study participants. Thus, the CART scale score was chosen as the dependent variable in the regression model, while the predictors included were the significance of the community, migration with someone or alone, and duration of stay in Italy (calculated in years). The change in adjusted R2 was calculated by removing all significant variables from the model. All tests were two-tailed, with *p* < 0.05 indicating statistical significance. Finally, a simple moderation analysis was carried out using the Macro Process for SPSS (48), with the perception of mental disorder as the independent variable and the perception of subjective well-being as the dependent variable. Within this relationship, perception of community resilience was included as a moderating variable. Moderation analyses allow us to analyze whether the relationship between the two variables is influenced by the values of a third variable. In this case, they allowed us to analyze whether the relationship between the perception of mental disorder and perceptions of subjective well-being varied according to different levels of perceived community resilience (i.e., the mean level and 1 SD above and below the mean).

## 3 Results

### 3.1 Descriptive statistics

The study included 100 patients with a migration background at the first consultation interview at the ambulatories of Psychiatry Unit, aged between 19 and 72 years old (M = 41.1, SD = 19.6). Among the participants, 54% were women. Most participants (40%) were married, had children (54%), attended high school (42%), and were employed (77%). Regarding psychiatric symptoms, 48% had anxiety symptoms, 37% depressive symptoms and 15% psychosomatic symptoms. Regarding migration, most participants (69%) had migrated with someone, primarily for work (42%) or family (38%) reasons, and the majority (37%) were from South America. Finally, most participants (63%) attributed the meaning of the host community to the word “community”, arrived in Italy between 1960 and 2021, and had spent an average of 19 years in the host country. The mean scores were 133 for subjective well-being (SD = 38.4, range = 42-210), 87.6 for perceived community resilience (SD = 21, range = 40-120) and 10.8 for mental disorder (SD = 5.1, range = 2-20). Descriptive statistics and correlation analyses are shown in **Tables 1**, **2**.

**Table 1:** Descriptive Statistics.

		*N*	*%*
Gender			
	Female	54	54%
	Male	46	46%
Marital Status			
	Married	40	40%
	Separated	18	18%
	Widower	1	1%
Education level			
	Primary school	6	6%
	Middle school	28	28%
	High school	42	42%
	Graduation	28	28%
Mental Disorders			
	Depressive symptoms	37	37%
	Psychosomatic symptoms	15	15%
	Anxiety symptoms	48	48%
Solitary migration			
	Yes	31	31%
	No	69	69%
Meaning attributed to the word “community”			
	Ethnic community	37	37%
	Host Community	63	63%
		*M*	*SD*
**Age (in years)**		41.1	19.6
**Duration of stay in Italy (in years)**		19	14
**Subjective Wellbeing**		133	38.4
**Perceived Community Resilience**		87.6	21.0
**Psychopathology**		10.8	5.1

**Table 2:** Correlation Matrix.

		SRQ24		CART_T		I_COPPE
SRQ24	Pearson r	—				
	gdl	—				
	*p* value	—				
CART_T	Pearson r	-0.232	*	—		
	gdl	98		—		
	*p* value	0.020		—		
I_COPPE	Pearson r	-0.459	***	0.429	***	—
	gdl	98		98		—
	*p* value	< .001		< .001		—

*p <.05, **p <.01, ***p <.001; SRQ24 = Total score of Self reporting questionnaire; CART_T = Total score of Communities Advancing Resilience Toolkit Assessment Survey – Core Resilience; I_COPPE = Total score of Interpersonal, Community, Occupational, Physical, Psychological and Economic Wellbeing Scale.

### 3.2 Simple linear regression

The simple linear regression equation was statistically significant, F(3,96) = 22.151, *p* < 0.001, for the variance in the perception of community resilience (R² = 0.41, adjusted R²= 0.39). **Table 3** shows the multicollinearity test. **Table 4** shows that the attribution of the host community’s meaning to the word “community”, having migrated with someone (i.e., not solitary), and duration of stay (in years) in the host context explained 39% of the variance. The linear regression model was tested only on the community resilience variable and did not consider the other variables analyzed in the correlation because the objective of the study was to explore which variables promoted community resilience (see *Hypothesis 1*).

**Table 3:** Collinearity statistics.

	VIF	Tolerance
Duration of stay (in years)	1.10	0.905
Migration with someone	1.18	0.845
Ethnic community	1.82	0.548
Host community	1.90	0.526

**Table 4:** Coefficients of predictors of perceived community resilience.

Predictors	B	SE	t	*p*	*B*
Intercept	66.010	3.285	20.096	< .001	
Host community	17.168	3.462	4.959	< .001	0.414
Migration with someone	11.135	3.573	3.116	0.002	0.263
Duration of stay (in years)	0.331	0.119	2.778	0.007	0.223

### 3.3 Moderation analysis

The moderation analysis shown in **Table 5** reveals that there is a statistically significant interaction between presence of mental disorder and perceived community resilience [b = 0.05, SE = 0.02, 95% C.I. (0.00, 0.10), *p* = .04]; that is, perceived community resilience had a moderating effect on the relationship between perception of mental disorder and perception of subjective wellbeing. The results highlight that there is also a direct negative relationship between mental disorders and subjective well-being. Specifically, when there was a high perception of mental disorders, there was a significant decrease in the perception of subjective well-being [b = -2.84, SE = 0.61, 95% CI (-4.04, -1.64), *p* <.001].

**Table 5:** Moderation Analysis.

			95% Confidence Interval			
	*B*	SE	Lower	Upper	Z	*p*
Main Effect						
Mental disorder	-2.8432	0.6122	-4.0432	-1.6433	-4.64	< .001
Perceived community resilience	0.5598	0.1477	0.27038	0.8492	3.79	< .001
Interaction Effect						
Mental disorder X Perceived community resilience	0.0506	0.0251	0.00155	0.0997	2.02	0.043

**Figure 2:**
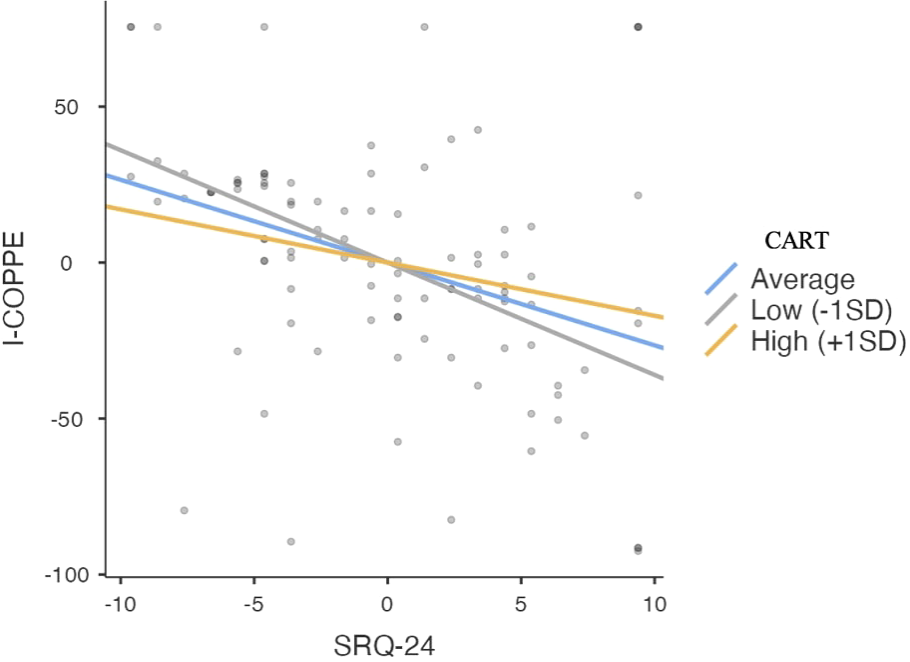
Simple Slope Analysis Plot. SRQ-24 = Total score of Self reporting questionnaire; CART = Total score of Communities Advancing Resilience Toolkit Assessment Survey – Core Resilience; I-COPPE = Total score of Interpersonal, Community, Occupational, Physical, Psychological and Economic Wellbeing Scale.

Looking at the results of the simple slope analysis (**Table 6**), one can see how the effect of the predictor (i.e., perception of mental disorders) varies its effect on the dependent variable (i.e., perceived subjective well-being) at different levels - all statistically significant (*p* <.05) - of the moderating variable, which was the level of perceived community resilience. In particular, as shown in **Figure 2**, when perceived community resilience was below average (-1SD), the perception of mental disorders led to a much greater decrease in subjective well-being [b = -3.90, 95% CI (-5.45, -2.35), *p* <.001] than when perceived community resilience was above average (+1SD). In this case, the perception of mental disorder still led to a decrease in subjective well-being, but to a lesser extent [b = -1.78, 95% CI (-3.43, -0.13), *p* = .03] (**Figure 2**).

**Table 6:** Simple Slope Analysis.

			95% Confidence Interval			
	*B*	SE	Lower	Upper	Z	*p*
Average	-2.84	0.621	-4.06	-1.625	-4.58	< .001
Low (-1SD)	-3.90	0.790	-5.45	-2.355	-4.94	< .001
High (+1SD)	-1.78	0.842	-3.43	-0.131	-2.12	0.034

## 4 Discussion

The aim of this study was to explore for the first time that which variables in migration predicted increased perceived community resilience and to assess the role of community resilience in the relationship between mental disorder and subjective well-being, in a sample of migrants at the first consultation interview in the ambulatories of the Psychiatry Unit.

Assessing participants’ mental disorders using the SRQ-24 questionnaire, it was found that 37% reported depressive symptoms, 48% anxiety symptoms and 15% psychosomatic symptoms. These findings are crucial for understanding how various psychiatric conditions can affect community resilience and the subjective well-being of migrants. Depressive symptoms, such as loss of interest, sadness, and negative self-perception, can significantly hinder migrants’ adaptation to the host context, making it difficult for them to access social support and community resources (49). Anxiety symptoms, which affect almost half of the participants, can influence risk perception and response to uncertainty, potentially limiting integration and support network development. High levels of anxiety may also contribute to social isolation, a critical factor for community resilience that is promoted by feelings of connection to the community (36). Psychosomatic symptoms, found in 15% of the participants may reduce participation in the community and in activities to promote integration.

In relation to the first hypothesis, which was confirmed through simple linear regression analysis, the results show that attributing community meaning to the receiving context, migrating with someone, and spending more time in the host context are key variables in predicting an increase in perceived community resilience. In migration, a community can take on multiple meanings: migrants can refer to both their ethnic community and that of the host context, which may be a key factor in perceived community resilience (16). The attribution of the meaning of the community to that of the host context could be representative of a sense of inclusion and integration in that context. Feeling integrated into the context may facilitate access to resources, social support, and information, about medical care services in the area, and these factors contribute to perceptions of community resilience, in line with previous studies (50). Migrating with someone, as a factor in promoting community resilience, may have several explanations. First, embarking on the migration journey with someone can foster the creation of networks that can provide social, emotional, or material support, as well as the sharing of practical information, including information about health services in the host country, which are key to promoting community resilience. In addition, migration sharing can enable the sharing of financial and material resources, such as living spaces, and the creation of informal work networks. Sharing spaces with people of the same ethnicity and who speak the same language fosters a sense of home (51), and in addition being able to work in the host context can foster a sense of pride and a feeling of contributing to the economic growth of the host country; these factors are promoters of community resilience, as previous studies have shown (52). Finally, migrating with others of the same ethnicity could foster the ability to maintain a connection with one’s culture of origin, thus preserving one’s ethnic-cultural identity and promoting community resilience. In line with this, a recent study (53) found that migrants, having a flexible approach to the host culture while preserving one’s culture of origin is indeed a factor in promoting community resilience. With respect to length of stay in Italy, spending more time in the Italian host context was found to be a significant predictor of increased community resilience. This finding contrasts with previous studies (54) that found in longitudinal studies that community resilience decreases over time following disasters, such as the Covid-19 pandemic. Indeed, community resilience has been studied in the literature following far-reaching events such as environmental disasters or pandemics, while it is less studied in relation to slow-onset stressors, such as migration (55). However, if in relation to far reaching events, community resilience may decrease over time due to, for example, the depletion of resources available to cope with that event but also to the reduced activation of informal or formal support networks over time (56); in contrast in relation to migration, community resilience may increase over time for different reasons. Once they arrive in the host country, migrants go through different stages and over time access the re-establishment phase (10) that allows migrants to feel that context as their own. This could be reflected, as found in a recent study (57), in a greater sense of being able to access resources in the host context or to have acquired more information about the bureaucracy or services of the context, and which are factors that promote community resilience.

Regarding the second hypothesis, which was confirmed through moderation analysis, our results show that there is a significant relationship between mental disorders and subjective well-being in the sample of migrants. Specifically, the perception of mental disorders had a direct negative effect on subjective well-being by reducing it, as demonstrated in other studies that report how the presence depressive or anxious symptoms reduces the perception of well-being (21, 58, 59). In fact, the presence of psychopathological symptoms can affect well-being, participation, and interactions within the host community, leading to social isolation and less participation in the community, thus reducing the sense of belonging and perception of social support (60). Finally, the perception of mental disorder can lead to reduced job satisfaction, difficulty in coping with challenges in the workplace, and in some cases, loss of employment itself, resulting in a negative impact on subjective well-being (61). Confirming Hypothesis 2, the results also show that perceived community resilience moderates the relationship between mental disorder and well-being and mitigates the impact of mental disorder on migrants’ subjective well-being. Specifically, the results show that when perceptions of community resilience are lower than average (i.e., when people perceive that their community is less resilient), the perception of mental disorder has a significantly greater impact on decreasing subjective well-being. However, when the perception of community resilience is higher than average (i.e., when people perceive that their community is more resilient), the perception of mental disorder still has a negative impact on subjective well-being, but to a lesser extent. This finding underscores the importance of perceived community resilience in mitigating the negative effects of mental disorders on subjective well-being. Perceiving one’s community as more resilient seems to provide a kind of “protection” against the effects of mental disorders on subjective well-being. This result is in line with previous study (40) in which researchers investigated 2246 adult participants and found that community resilience moderated the relationship between depressive and anxiety symptoms and quality of life. Perceiving the community as resilient may reflect migrants’ feelings of support and connections with other community members. This can help mitigate feelings of isolation and loneliness associated with mental disorders, and thus reduce their negative impact on subjective well-being. It may also reflect the perception of having access to community resources and mental health services that can help address psychopathology and the information required to access these services. Finally, it may relate to the feeling of agency within the community, that is, having an active role within the community and not being passive toward mental suffering.

In conclusion, it is important to emphasize that several factors may play a role in the relationship between well-being, community resilience and mental disorders, including affective temperaments. In fact, temperaments, defined in terms of variations in the expression of core psychic components, such as psychomotor, thinking, and mood (60), can influence perceptions of and access to community resources. In addition, affective temperaments could influence individual and social interactions within a community, thus contributing to the construction of specific community dynamics.

For example, individuals with a depressive temperament might experience difficulties in adapting to change and finding confidence in community resources, whereas those with a hyperthymic temperament might actively contribute to the building of strong social networks and the promotion of a culture of resilience based on collaboration, mutual trust, and facing the challenges of migration with determination. Understanding such individual aspects could shed light on the link between individual and community factors in perceived community resilience as demonstrated by a recent study (33). Future studies should investigate the relationship between these aspects by highlighting the role of affective temperaments.

The results of this study offer valuable insights and significant practical implications. They underscore the critical importance of adopting a community-based approach to address the psychopathology of migration. Implementing community resilience factors is essential for managing migrants’ mental health challenges. Psychiatric services can play a pivotal role in bridging gaps within the community, supporting migrants and facilitating their access to essential services, information, and community resources. By promoting these resilience factors, interventions can more effectively mitigate the impact of psychopathology on the subjective well-being of migrant populations, thereby enhancing their overall mental health and integration into the host society. Moreover, these findings can inform the clinical practices and policies aimed at supporting migrants. Clinicians can leverage these insights to design culturally sensitive therapeutic interventions that bolster community resilience, thereby enhancing patients’ well-being. Psychiatric services can also play a bridging role in the community and support migrants in accessing services, information, and community resources that are useful for migration. Finally, understanding these specific psychiatric conditions allows for personalized support strategies that address different needs and vulnerabilities and this approach ensures that customized interventions regarding community resilience factors are promoted.

Policymakers can use this evidence to develop programs that facilitate community integration, provide social support, and ensure migrants have access to essential resources and services. By addressing community-level factors, such initiatives can create a more supportive environment that promotes migrants’ mental health and overall well-being. In conclusion, through collaborative efforts among healthcare providers, policymakers, and community stakeholders, the implementation of these policies can facilitate the successful integration of migrant populations and contribute to their flourishing within the host society.

## 5 Limitation of the study

Although this study provides valuable insights into the relationship between perceived community resilience, mental disorder, and subjective well-being among migrant patients, it has several limitations that need to be considered. This study used a convenience sample of adult migrants at the first consultation interview in the ambulatories of Psychiatry Unit. This sample may not be representative of all migrant populations as it includes only individuals seeking psychiatric care in a hospital setting. The results may not be generalizable to migrants living in the community or to those not seeking mental health treatment. The study’s inclusion criteria may have introduced a selection bias. The exclusion of individuals who did not understand Italian may have excluded some migrant groups, potentially distorting the results. This study used a cross-sectional design, which provided only a snapshot of the data at a single point in time. This design cannot establish causality or determine the direction of observed relationships. Considering this limitation, it is important to consider alternative interpretations of the results. Although this study shows an association between perceived community resilience and subjective well-being, causality cannot be established. Indeed, it is plausible that more severe psychiatric symptoms may decrease subjective well-being and perceived community resilience in several ways. First, the severity of such symptoms may contribute to widespread distress, thereby reducing the overall level of perceived well-being. Additionally, severe psychiatric symptoms may affect the perception of community resilience. Individuals facing severe psychiatric symptoms may perceive their community as being less able to provide support and resources to cope with challenges. This perception may be amplified by feelings of social isolation or a lack of support, leading individuals to doubt their community’s ability to cope.

Longitudinal studies are needed to better understand how perceived community resilience affects the relationship between perception of mental disorder and subjective well-being over time. Finally, the collected data were based on self-reported measures, which are subject to biases such as social desirability and recall bias. Participants may have provided responses perceived as socially desirable or may have had difficulty accurately recalling their perceptions and experiences. Future research should address these limitations to provide a more comprehensive understanding of the factors that influence mental health outcomes in migrant populations.

## 6 Conclusion

This study provides preliminary evidence of the role of community resilience as a protective factor against the effects of mental disorder on well-being in a sample of migrants at the first consultation interview in the ambulatories of the Psychiatry Unit. Our results support an ecological model of migrants’ mental health by evaluating the community and its resources in the management of psychopathology in the context of migration. Activating community resilience promotion programs can be a valuable addition to the traditional therapeutic approaches. Future research should explore several areas to deepen our understanding of community resilience in migrant populations. Longitudinal studies could provide insights into how community resilience evolves over time and its long-term effects on mental health. Investigating the specific components of community resilience that are most beneficial for different migrant groups could help to tailor interventions more effectively. Finally, comparative studies across different countries and cultural contexts could shed light on the universal versus context-specific aspects of community resilience, informing globally applicable strategies for supporting migrant mental health. In conclusion, policymakers should recognize the role of community resilience factors in contributing to migrant well-being, and thus be able to contribute to the implementation of multilevel interventions in which hospital services can play a central role in facilitating access to these community interventions.

## Data availability statement

The raw data supporting the conclusions of this article will be made available by the authors upon reasonable request. Requests to access the datasets should be directed to francesco.madera@edu.unige.it.

## Ethics statement

The studies involving humans were approved by the Ethical Research Committee of the University of Genoa. The studies were conducted in accordance with the local legislation and institutional requirements. The participants provided their written informed consent to participate in this study.

## Author contributions

MO: Conceptualization, Methodology, Writing – original draft, Writing – review & editing. FM: Formal analysis, Methodology, Writing – original draft, Writing – review & editing, Conceptualization. PC: Conceptualization, Project administration, Supervision, Writing – original draft, Writing – review & editing. GS: Conceptualization, Project administration, Writing – original draft. LM: Conceptualization, Project administration, Supervision, Writing – original draft, Writing – review & editing.
